# Biomechanical influence of the surgical approaches, implant length and density in stabilizing ankylosing spondylitis cervical spine fracture

**DOI:** 10.1038/s41598-021-85257-8

**Published:** 2021-03-16

**Authors:** Yaoyao Liu, Zhong Wang, Mingyong Liu, Xiang Yin, Jiming Liu, Jianhua Zhao, Peng Liu

**Affiliations:** 1 Department of Spine Surgery, Army Medical Center of PLA (Daping Hospital), Army Medical University, No. 10 Changjiang Road, Yuzhong District, Chongqing, 400042 China; 2 China Jiejun Technique Corporation, Chongqing, China

**Keywords:** Diseases, Medical research

## Abstract

Ankylosing spondylitis cervical spine fractures (ASCFs) are particularly unstable and need special consideration when selecting appropriate internal fixation technology. However, there is a lack of related biomechanical studies. This study aimed to investigate the biomechanical influence of the pattern, length, and density of instrumentation for the treatment of ASCF. Posterior, anterior, and various combined fixation approaches were constructed using the finite element model (FEM) to mimic the surgical treatment of ASCFs at C5/6. The rate of motion change (RMC) at the fractured level and the internal stress distribution (ISD) were observed. The results showed that longer segments of fixation and combined fixation approaches provided better stability and lowered the maximal stress. The RMC decreased more significantly when the length increased from 1 to 3 levels (302% decrease under flexion, 134% decrease under extension) than from 3 to 5 levels (22% decrease under flexion, 23% decrease under extension). Longer fixation seems to be more stable with the anterior/posterior approach alone, but 3-level posterior fixation may be the most cost-effective option. It is recommended to perform surgery with combined approaches, which provide the best stability. Long skipped-screwing posterior fixation is an alternative technique for use in ASCF patients.

## Introduction

Ankylosing spondylitis (AS) is a kind of chronic, systemic and inflammatory rheumatic spondyloarthropathy involving mainly the sacroiliac joints and spine, with an overall prevalence of 0.1–1.4%^1–7^. Severe spinal instability following fractures can be caused even in cases of minor trauma due to paravertebral ligament and intervertebral disc ossification, poor bone quality and long lever arms. The most common fracture site is the cervical spine, especially the subaxial cervical spine^8–11^. Ankylosing spondylitis cervical spine fractures (ASCFs) are particularly unstable, as they typically involve both the ventral and dorsal elements and typically occur at the level of intervertebral disc ossification^8,10,12–14^. Nonsurgical treatments, such as treatment with a cervicothoracic brace, halo vest and axial traction^2,4,15–19^, cannot be suggested due to the unacceptably high incidence of complications^19,20^, including union failure, increased neurological deficit or death^19^. Surgical treatments appear to produce immediate stability and avoid the need for prolonged bed rest for cervical traction and immobilization. Meanwhile, it seems to be the only viable way to reconstruct dislocated vertebral bodies and decompress the spinal cord and nerves, which could provide conditions for functional recovery^2^. Studies have shown that among those who do not undergo surgical stabilization, as many as 60% go on to develop progressive neurological deficits due to secondary dislocation at the fractured level^6,21,22^. Therefore, surgical treatment is an appropriate therapeutic method for ASCF patients.

Surgical approaches for ASCF include anterior spinal fusion (ASF)^23,24^, posterior spinal fusion (PSF)^11,25–27^, and anterior–posterior spinal fusion (APSF)^28–32^, each of which achieves partial therapeutic success. However, a consensus regarding the surgical approach and implant length and density has not been achieved, as the present available literature mainly consists of low-level evidence case series or case reports with small samples^23,27,30^. To our knowledge, there is a lack of related biomechanical studies. This study aimed to investigate the biomechanical influence of the length and density of instruments for the treatment of ASCF using finite element testing.

## Materials and methods

### Simulation of AS

**Figure 1:**
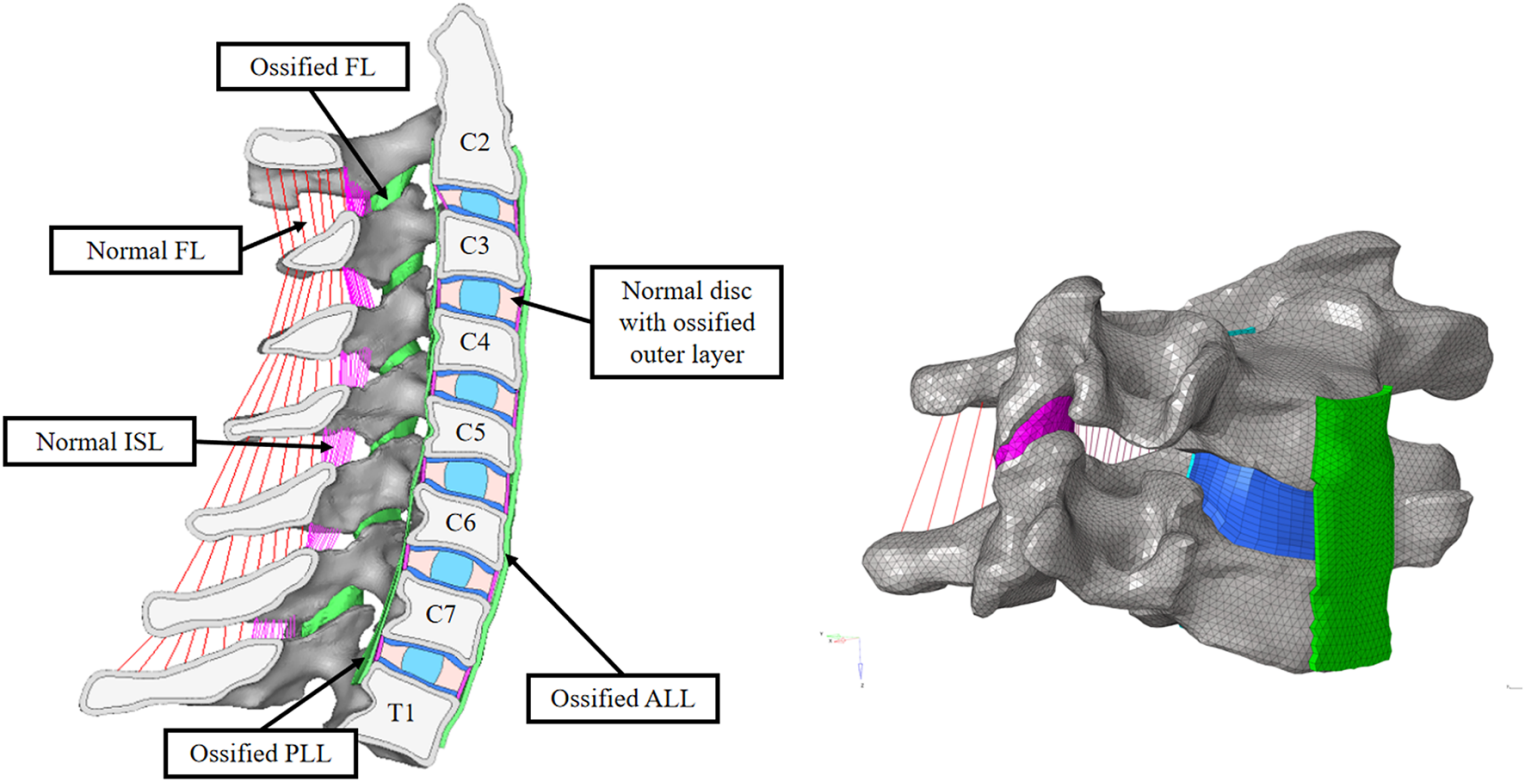
Finite element model used for the simulations in this study. Color coding was used to distinguish the different parts of the model. Ligaments (ALL/PLL/CL) (green) and the outermost layer of the annulus are defined as sites of ossification to simulate the end stage of AS in the cervical spine.

In this study, a C2–T1 AS model was adjusted based on the previously developed and validated finite element model (FEM) to simulate the ligamentous subaxial cervical spine^33,34^. Different loading mechanisms have been compared at the level of the functional unit, including under flexion and extension. The specific features of late-stage AS are as follows: (1) the outer layer of the intervertebral disc, posterior longitudinal ligament (PLL), anterior longitudinal ligament (ALL), and capsular ligament (CL) are ossified^35^; (2) there is osteoporosis of the spine, especially the vertebral body^36^; (3) the upper cervical spine is usually functional, while the caudal end of the cervical spine is relatively fixed due to thoracic and heterotopic ossification^37,38^; (4) paravertebral muscles provide active stability^38^; and (5) there is sometimes excessive lordosis in the subaxial cervical spine and kyphotic deformity of the cervicothoracic region^39^. To simulate the above characteristics, the initial model was modified as follows: (1) the outermost layer of the annulus, ALL, PLL and CL were remodeled by changing the material properties from those of soft tissue to those of newly formed bone, which are similar to those of cortical bone; (2) We measured the thickness of ossification of outmost layer of annulus, ALL and PLL from AS patients. Outmost layer of annulus and PLL is among 0.37–0.67 mm and ALL is among 0.60–1.20 mm. We chose 1 mm for ALL and 0.5 mm for PLL and outmost layer of annulus the simulation at last; (3) the vertebrae were considered rigid, while the interspinous ligament (ISL) and ligamentum flavum (FL) remained soft; and (4) the material properties of osteoporotic cortical/cancellous bone and ossified bone were adjusted according to the literature^40–42^ (Fig. 1). Material properties^43–50^ are listed in Table 1.

**Table 1:** Material properties assumed for initial model and AS model.

Name	Element type	Material model	Material property	References
Cortical bone	C3D4	Isoelastic	E = 8000 MPa μ = 0.3	Wheeldon et al.^43^
Cancellous bone	C3D4	Isoelastic	E = 100 MPa μ = 0.3	Wheeldon et al.^43^
Newly formed bone	C3D4	Isoelastic	E = 3500 MPa μ = 0.3	Vosse and Vlam^36^
Cartilaginous end-plate	C3D8	Isoelastic	E = 23.8 MPa μ = 0.3	Schmidt et al.^47^
Cartilage of joint	C3D8	Isoelastic	E = 23.8 MPa μ = 0.3	Schmidt et al.^47^
Nucleus	C3D8H	Hyperelastic	C10 = 0.12, C01 = 0.09	Schmidt et al.^47^
Annulus ground substance	C3D8H	Hyperelastic	C10 = 0.133, C01 = 0.0333, D = 0.6	Kallemeyn et al.^75^
				Leahy et al.^76^
Annulus fiber	SpringA	Nonlinear spring	Stress–strain curve	Shirazi-Adl et al.^50^
				Holzapfel et al.^45^
				Schmidt et al.^47^
Instrumentation	C3D8	Isoelastic	E = 110,000 MPa μ = 0.3	Hussain et al.^44^
Cage	C3D8	Isoelastic	E = 3500 MPa μ = 0.3	Hussain et al.^44^
ligaments	SpringA	Nonlinear spring	Force–deflection curve	Yoganandan et al.^46,48^

***E***, Young modulus; *μ*, Poission’s ratio; Isoelastic, isotropic.

### Fracture modeling

**Figure 2:**
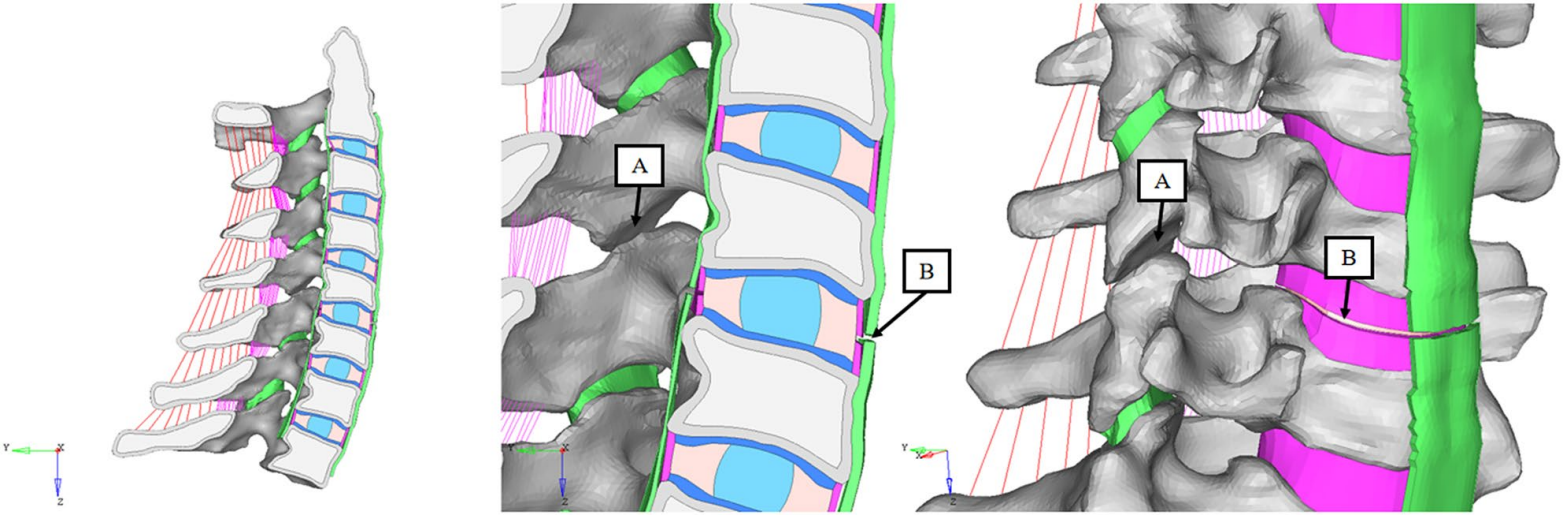
AS cervical fracture model used in this study. The whole CL was removed, and 0.5 mm of the ALL and PLL was removed. (**A**) Fracture of CL. (**B**) Fracture of the outer layer of the annulus, ALL and PLL.

ASCF mainly occurs in the subaxial cervical spine, predominantly at C5–C7^3,4,8,10,11,51,52^, typically at the level of intervertebral disc ossification^8,10,12–14^. Therefore, a fracture at the C5–C6 disc level was modified from the basic intact model by removing the ossification of the disc and articular capsule. The whole posterior CL was removed. Then, 0.5 mm of the ALL and PLL was also removed. This means that only the inner disc, FL and ISL remained connected in the C5–C6 intervertebral interspace. No dislocation was simulated in this study (Fig. 2).

### Implant modeling

**Figure 3:**
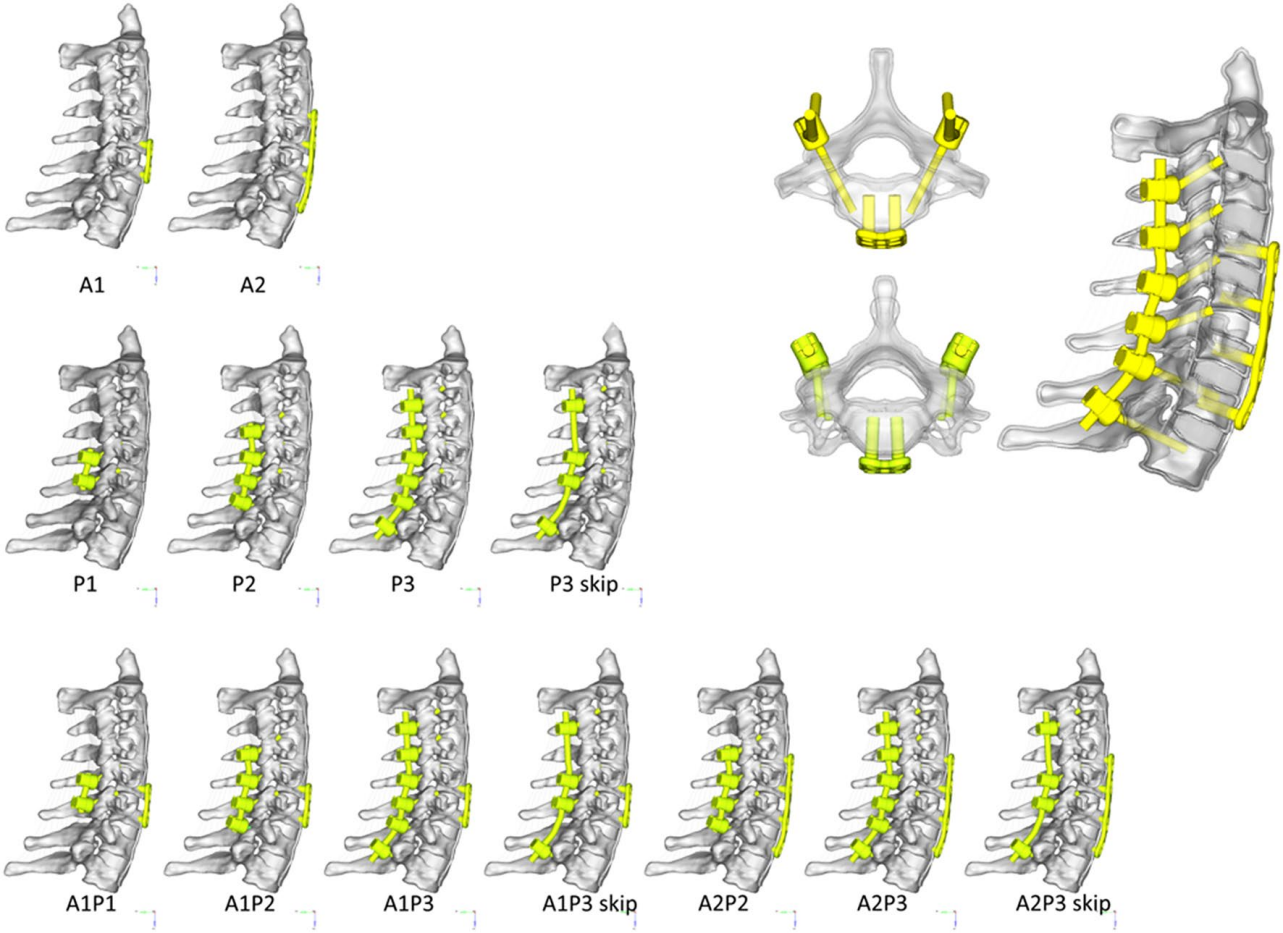
Lateral view of the thirteen implant configurations and schematic diagram of the implants.

Three fixation techniques, ASF, PSF and APSF, with different implant lengths and densities, were included in this study. Two models of anterior fixation alone were developed: 1-level fixation at C5–C6 (A1) and 3-level fixation at C4–C5–C6–C7 (A2). A titanium plate with a cage at C5/C6 was modeled in each anterior model. To simulate the posterior implant, lateral mass screws were used at C3/C4/C5/C6, while pedicle screws were used at C7/T1. Four kinds of posterior-alone fixation models were developed: 1-level fixation at C5–C6 (P1), 3-level fixation at C4–C5–C6–C7 (P2), 5-level fixation at C3–C4–C5–C6–C7–T1 (P3) and skipped 5-level fixation at C3–C5–C6–T1 (P3 skip). Seven additional models of combined approaches were developed based on cross matching of the anterior and posterior models introduced above (A1P1, A1P2, A1P3, A2P2, A2P3, A1P3 skip, A2P3 skip). Different implant configurations chosen for evaluation and corresponding schematic diagrams are shown in Fig. 3.

In the model, screws and rods were simplified as cylindrical bars. Details of screws, including screw threads, were ignored, and screws were assigned elastic material properties (Table 1). The tip end of the pedicle screw was constrained to an element in the cancellous bone and that of the lateral mass screw was constrained to an element in the cortical bone. The head end of the screw was constrained to the cortical bone at the screw entrance point. This means that each screw was constrained to 2 points in the lateral mass or vertebral body. In the anterior approach, the screw head was then constrained to the plate. In the posterior approach, the screw head was constrained to the rod. The connection of the rod and screw rod did not allow rotation. Screws were implanted bilaterally for each vertebra regardless of the use of an anterior or a posterior approach. The anterior screws were 4.0 mm in diameter and 15 mm in length, and the posterior screws were 3.0 mm in diameter and 28 mm in length for pedicle screws and 18 mm in length for lateral mass screws. The meshing size is about 0.7 mm for soft tissue and bone, 0.25–0.5 mm for instruments.

### Loading and boundary conditions

In this study, to simulate in vivo daily activities, axial and follower loading were combined because this loading condition was reported to produce more realistic physiological kinematics^34,53^. First, a preload of 50 N from paravertebral muscles was applied to simulate muscle tension. Second, another axial load of 50 N perpendicular to the upper endplate of C2 was applied to simulate the weight of the head. Finally, a pure moment of 2 Nm was applied to the upper endplate of C2 to simulate flexion and extension motion.

### Software and hardware

Multiple spine surgeons manually segmented the original images, and Adobe Photoshop (Adobe Systems, California, USA) was used to reconstruct the anatomical model. The commercial software Amira (5.3.3, Visage Imaging, Carlsbad, CA) was used to check the quality of surface smoothing and segmentation. According to the acquired surface files, refinement and meshing were performed by HyperMesh (V12.0, Altair, Michigan, America). Abaqus (Simulia, Providence, RI) was selected to run the models.

### Output variables

The rate of motion change (RMC) at the fractured level and internal stress distribution (ISD) at the implant were chosen for evaluation of the different fixation configurations. The range of motion (RoM) was defined as the variation in the angle between C5 and C6 after loading. The RMC, a standardized value for evaluating the stability of the models, was determined as the RoM of the fixed segments minus that of the intact model divided by this value^53–55^. A negative value indicated that the stability of internal fixation was better than that of the initially intact model. However, a positive value indicated a decrease in stability. The principal maximal stress (MS) in the screws, plates and rods was used to analyze the stress concentration, which represented the point most likely to fail.

## Results

### Spinal kinematics

**Figure 4:**
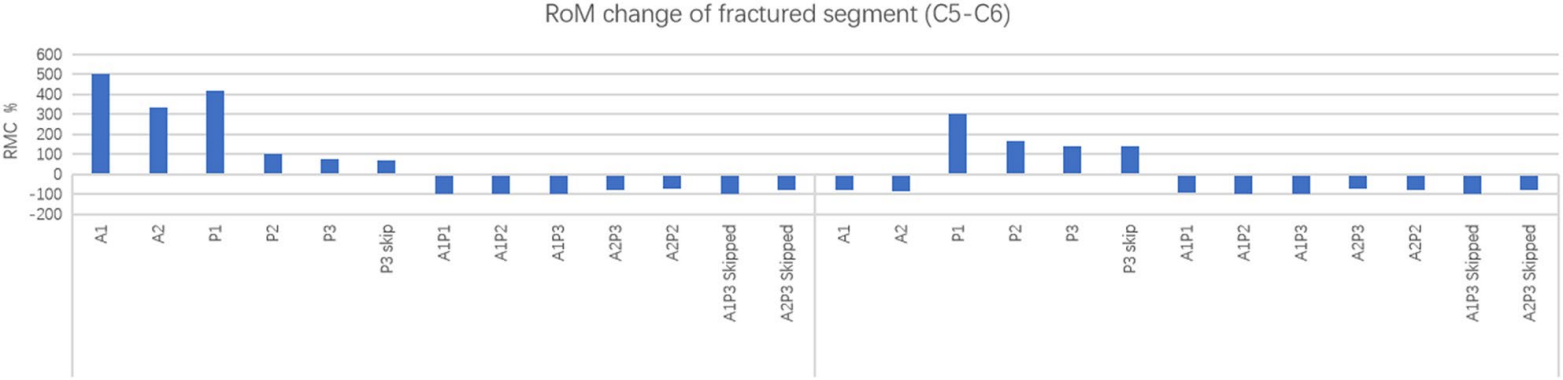
Sagittal RoM change at the fractured segment (C5/6) in different implant configurations.

In the intact AS cervical spine model, the RoM in the sagittal plane of segment C5/6 under loading was 0.8° under flexion and 0.13° under extension. The value served as a baseline for interpretation of the results in this section. Figure 4 shows the sagittal RMC of the fractured segment (C5/6) compared among all implant configurations after follower loading. Under flexion, the change in the RoM in models A1, A2 and P1 exceeded threefold, with a greater value for A1 (502%) than A2 (331%). In models P2, P3 and P3 skip, the change ranged from 58 to 69%. Note that the minimum RMC in all these combined APSF models was a negative value, which meant that these models were more stable than the intact model.

Under extension, the maximum RMC was 299% in P1. The value in the P2, P3 and P3 skip models was 165%, 142% and 141%, respectively. Models A1 and A2 and all combined APSF models presented negative values. The results indicate that ASF (A1 and A2) can resist extension but has no effect on resistance to flexion. Meanwhile, 1 level of PSF (P1) has no resistance to either flexion or extension; however, 3 or more levels of PSF (P2, P3 and P3 skip) show better stability than ASF and 1 level of PSF (P1). The most stable configurations may be the combined approaches, regardless of the mode of combined anterior and posterior fixation, including even the simplest 1-level combination (A1P1). Specifically, the sagittal RMC of P3 skip (141%) was not obviously higher than that of P3 (142%). This result might suggest that skipped screwing does not influence construct stability in posterior fixation.

### Stress analysis

**Figure 5:**
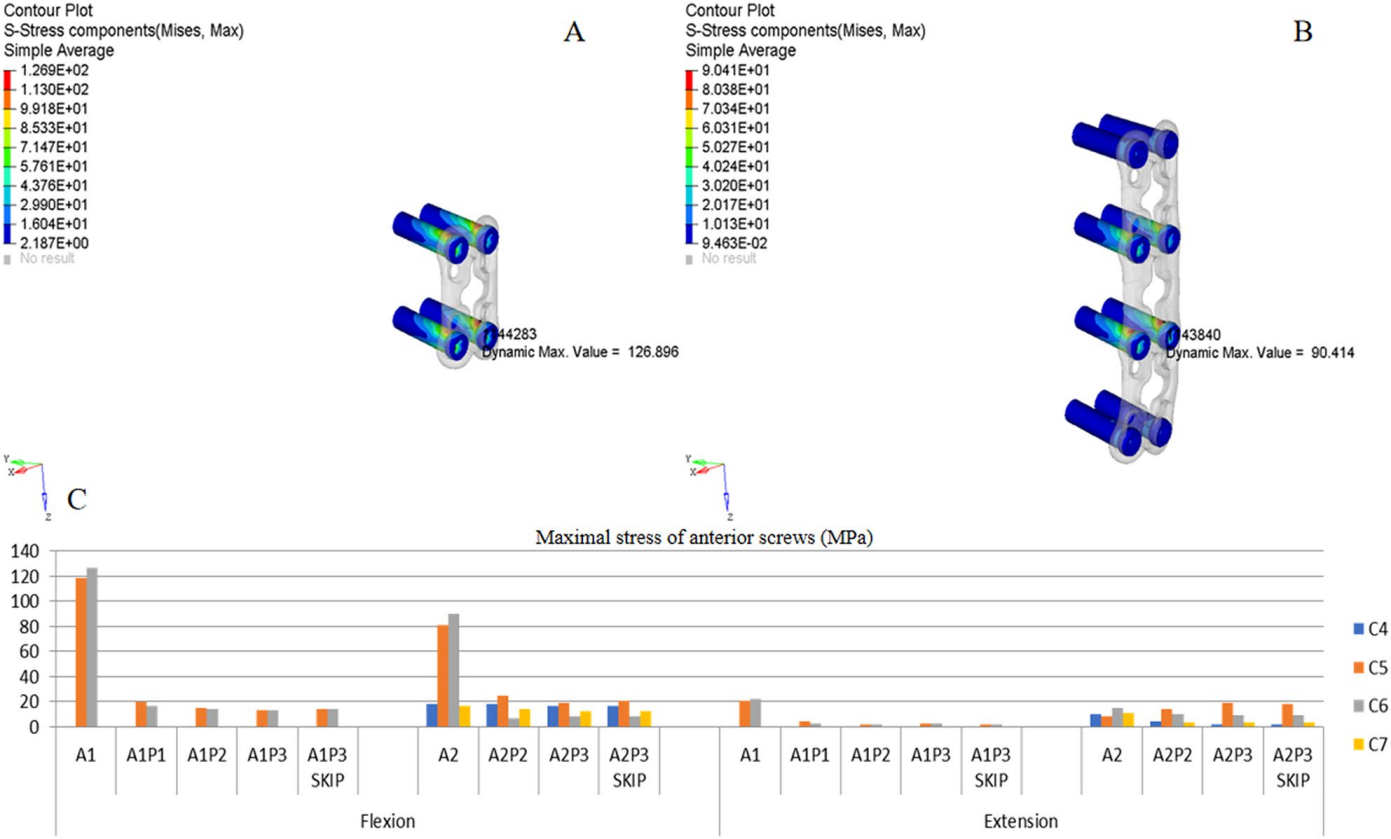
ISD of anterior screws. (**A**,**B**) Oblique view of von Mises stress distribution of the screws in A1 under flexion and A2 under extension. (**C**) MS of each anterior screw in different implant configurations.

**Figure 6:**
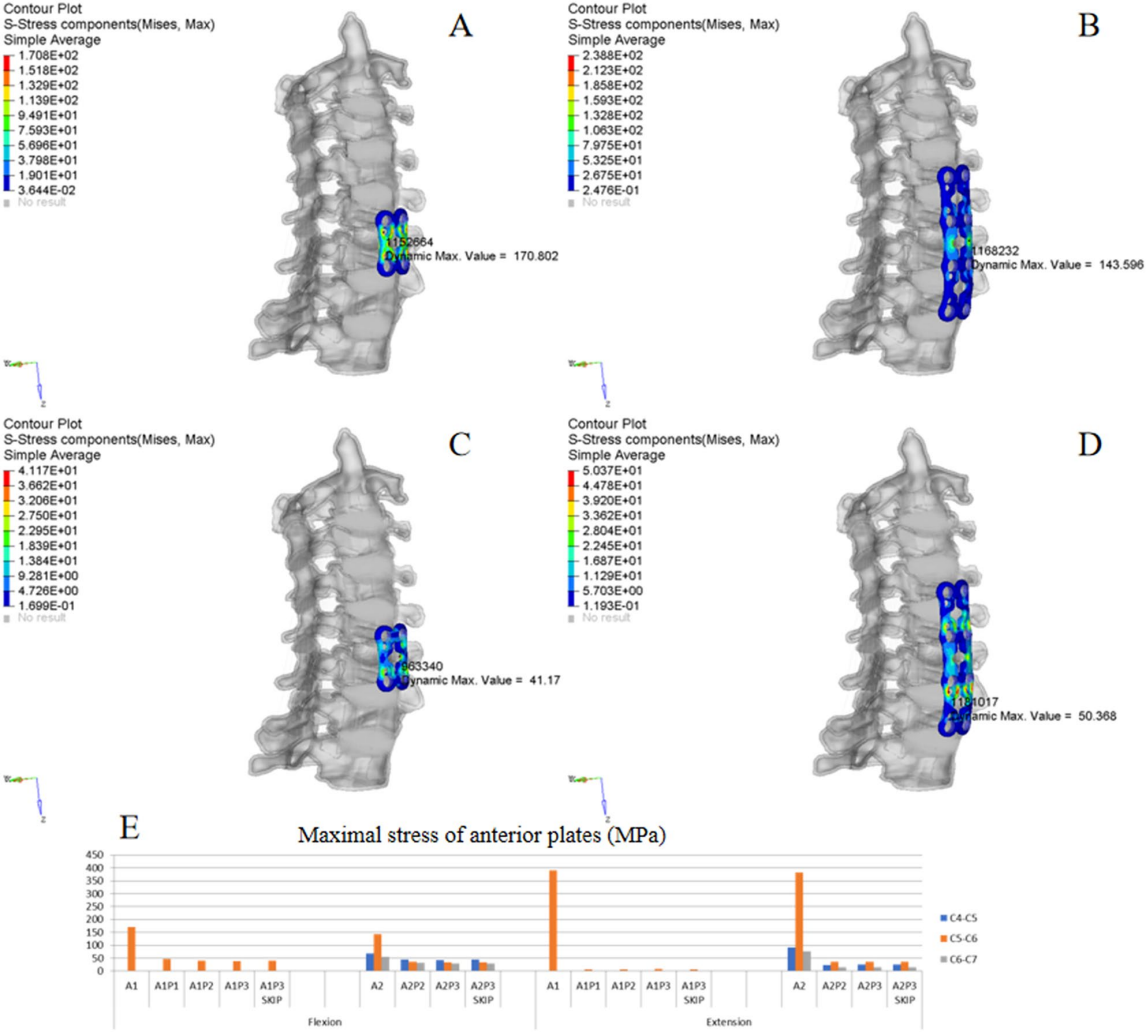
ISD of anterior plates. (**A**,**B**) Oblique view of the von Mises stress distribution of the plates in A1 and A2 under flexion. (**C**,**D**) Oblique view of the von Mises stress distribution of the plates in A1 and A2 under extension. (**E**) MS of plates in different implant configurations.

**Figure 7:**
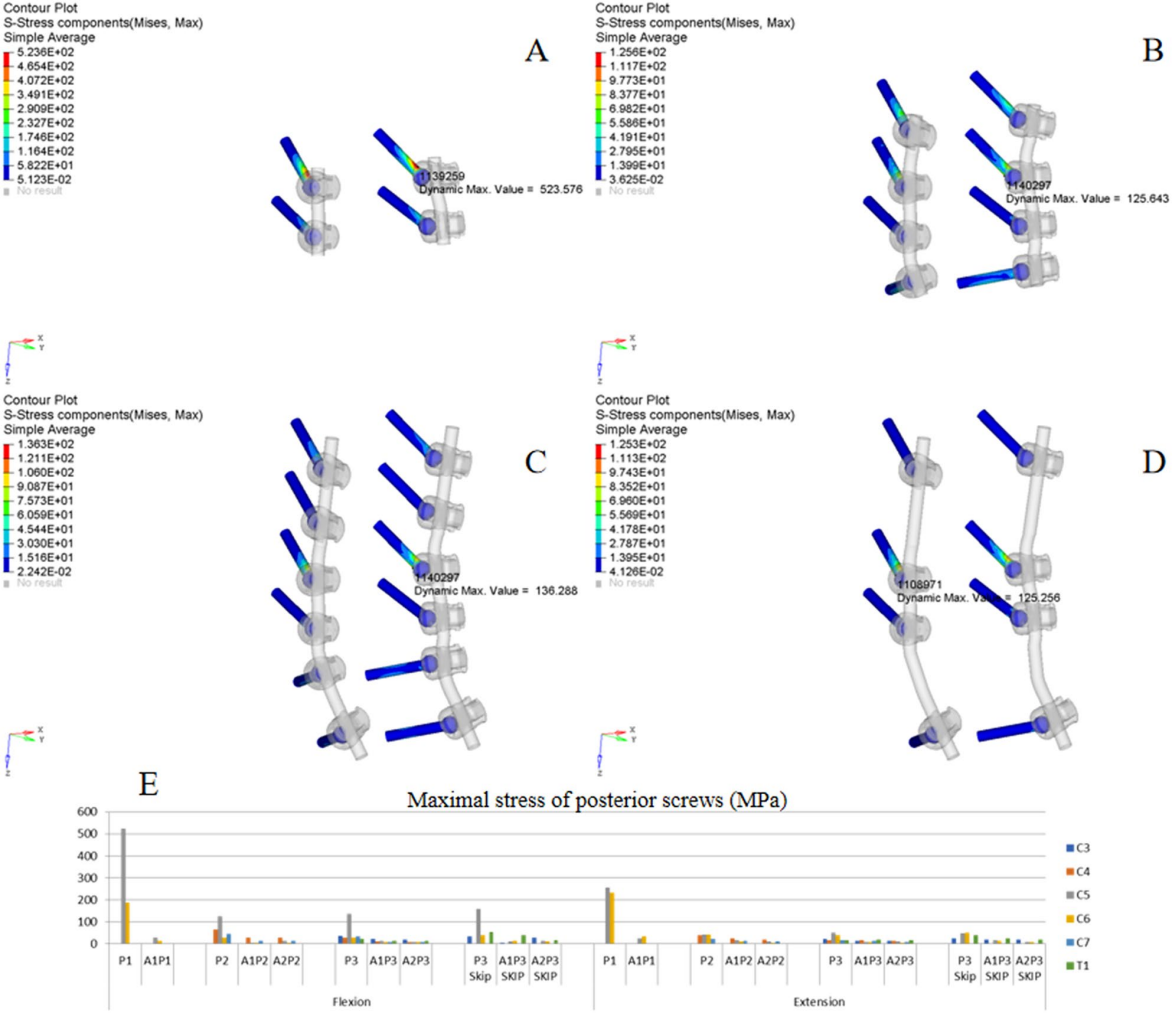
ISD of posterior screws. (**A**,**B**) Oblique view of von Mises stress distribution of the screws in P1 and P2 under flexion. (**C**,**D**) Oblique view of the von Mises stress distribution of the screws in P3 and P3 skip under extension. (**E**) MS of screws in different implant configurations.

**Figure 8:**
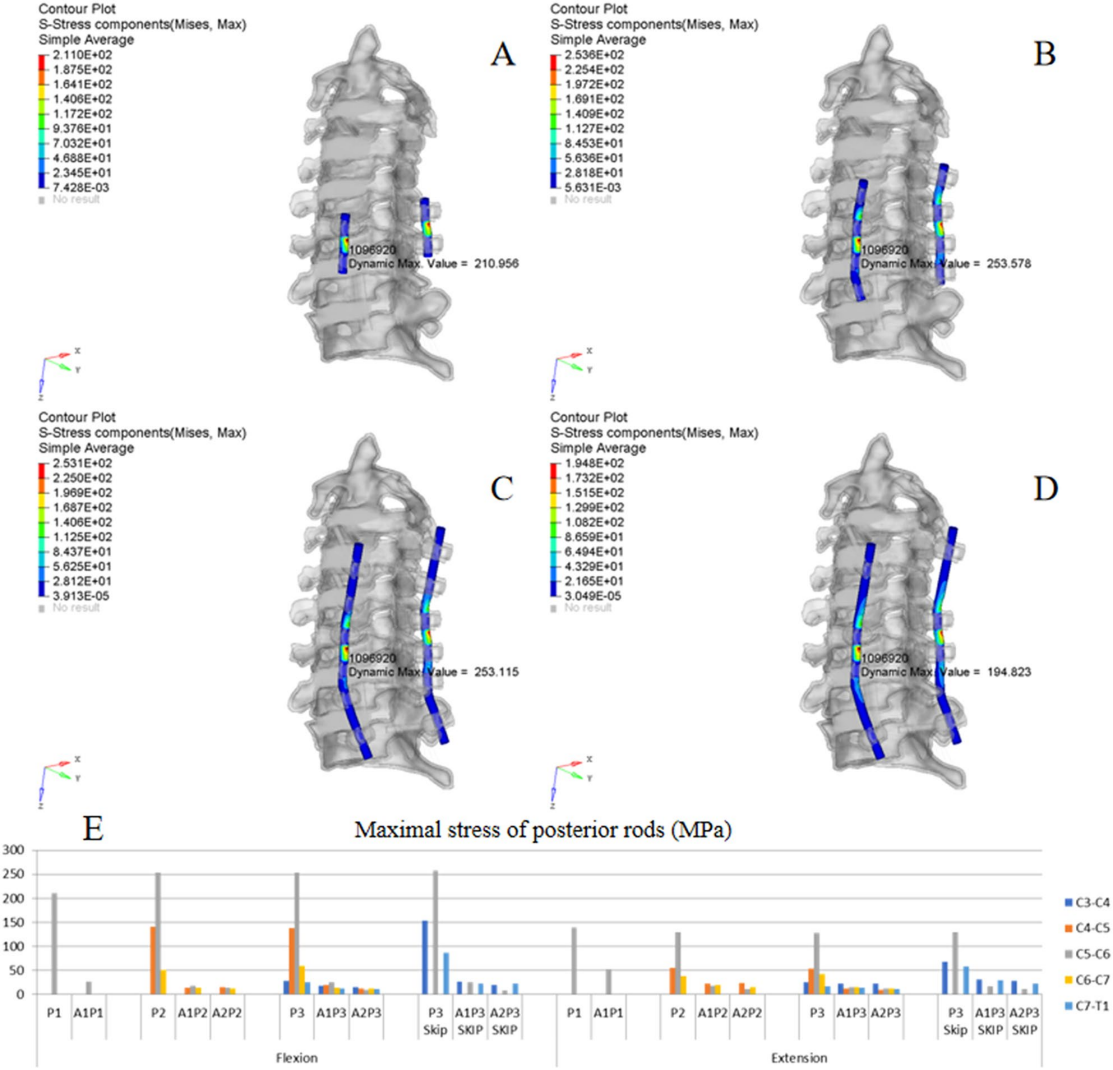
ISD of posterior rods. (**A**,**B**) Oblique view of the von Mises stress distribution of the rods in P1 and P2 under flexion. (**C**,**D**) Oblique view of the von Mises stress distribution of the rods in P3 and P3 skip under extension. (**E**) MS of rods in different implant configurations.

Figures 5, 6, 7 and 8 show the ISD and MS of anterior and posterior implants, including screws, plates, and rods. Both flexion and extension were simulated. All the results in this study show certain characteristics, as follows: (1) the stress was mainly concentrated at the head end of the screw, where the screw contacts the cortical bone or rod/plate; (2) regardless of the implant length, screws adjacent to the fractured segment presented the MS; and (3) stress on the plate/rod was mainly concentrated at the fractured level (C5/6).

### Anterior fixation

Figure 5A,B show the typical ISD of anterior screws under flexion (A1) and extension (A2), respectively. Figure 5C shows the MS of each anterior screw in different implant configurations. As presented in the histogram, the MS was mainly observed at the screw in C5 and C6. Comparing flexion and extension in all groups, the MS of the anterior screws under flexion was higher than that under extension. In the A1 model under flexion, the MS of C6 was 127 MPa, which was higher than the 90 MPa observed in the A2 model under flexion, indicating that increasing the internal fixation length could reduce the MS of the screw. Note that the combined fixation models could obviously reduce the MS of the anterior screw. All the stress values were less than 25 MPa, regardless of flexion or extension. Consequently, the difference in the MS among different combined fixation models was not obvious.

Figures 6A/B,C/D show the typical ISD of anterior plates under flexion and extension. Figure 6C shows the MS of each anterior plate in different implant configurations. The MS was mainly observed at the C5/6 level. In the A1 and A2 models, the MS was 390 MPa and 382 MPa under extension, compared with 171 MPa and 144 MPa under flexion. This trend was also shown by other implant configurations; in other words, the MS of the plate was higher under extension than under flexion. With increasing posterior fixation, the stress value decreased from 171 to 47 MPa, 41 MPa, 39 MPa and 39 MPa in the A1, A1P1, A1P2, A1P3 and A1P3 skip models, respectively. This result indicates that combined fixation could obviously reduce the MS of the anterior plate; however, increasing the fixation length did not significantly decrease the MS. In addition, skipped screwing had no significant effect on the stress distribution. Note that these trends were satisfied by A2 combined with PSF regardless of flexion or extension.

### Posterior fixation

Figure 7A/B,C/D show the typical ISD of posterior screws under flexion (P1/P2) and extension (P3/P3 skip), respectively. Figure 7E shows the MS of each posterior screw in different implant configurations. The MS was 523 MPa at C5 in P1 under flexion, which was significantly higher than that of P2 (125 MPa), P3 (136 MPa) and P3 skip (158 MPa), indicating that increasing the fixation length significantly decreases the MS of screws; however, the difference among P2, P3 and P3 skip were not significant. The trend was satisfied by posterior-alone extension and combined fixation models. In each fixation model, the MS of the screw was higher under flexion than under extension, as presented in the histogram; meanwhile, all the stress values in the combined fixation models were less than 45 MPa under flexion or extension, indicating that combined fixation could obviously decrease the MS of posterior screws, and the difference among the different combined fixation models was not obvious.

Figure 8A/B,C/D show the typical ISD of posterior rods under flexion (P1/P2) and extension (P3/P3 skip), respectively. Figure 7E shows the MS of each posterior rod in different implant configurations. The histogram indicates that the C5/6 level was the stress concentration source. Comparing different fixation lengths showed that increasing the fixation length did not significantly decrease the MS of the rod. The MS was higher under flexion than extension in the same fixation model. Meanwhile, compared with posterior-alone fixation, combined fixation could obviously decrease the peak stress of the rod, and the difference among different combined configurations was not obvious.

### Skipped screwing

As shown in Fig. 7E, the MS of the screw at C5 in P3 under flexion was 136 MPa compared 158 MPa in P3 skip; under extension, the value was 49 MPa compared 48 MPa. All the stress values in the combined fixation models (A1P3 vs. A1P3 skip and A2P3 vs. A2P3 skip) were less than 50 MPa under flexion or extension. Meanwhile, the MS of the rod at the C5/6 level was also less than 50 MPa in A1P3/A1P3 skip and A2P3/A2P3 skip (Fig. 8E). These results suggest that the effect of skipped screwing on the stress distribution of fixation was not significant.

## Discussion

To the author’s knowledge, the present study is the first biomechanical report comparing different fixation configurations and implant densities under quasi-static loading. As this study simulated instant postoperative stability, the results could facilitate clinical decision-making in cases of ASCF, especially with respect to the approach, fixation length and implant density.

Anterior plates fixed with screws and posterior plates or rods fixed with lateral mass or pedicle screws are the main surgical methods used for treating ASCF^11^. Regarding anterior approach technology, the risk of dislocation can be avoided due to the supine position assumed during the positioning process and the operation. Other obvious advantages of the anterior-alone approach are less trauma, fewer soft tissue complications, direct decompression of anterior compression, and a high interbody fusion rate; studies have reported many successful cases^23,24^. Kouyoumdjian^23^ reported that 16 patients were successfully cured. However, ASCF commonly involves three columns of the spine, leading to severe instability, which may render anterior-alone fixation unreliable. The fixation failure rate was 50% among patients treated with this approach^56^. Our results also confirm that the anterior-alone approach with the largest RMC was the most unstable method compared with long posterior and combined fixation approaches. This approach provides little resistance to flexion, meaning fixation failure may occur during cervical flexion. In fact, the anterior approach for treating ASCF is still unpopular among surgeons; only 15% of ASCF cases were treated with this method alone^57^. The present study indicates that the longer the anterior fixation level, the smaller the RoM change, which suggests higher stability in resisting long movement arms. Thus, for those ASCF patients who can only undergo ASF, such as those with excessive cervical lordosis or technical limitations, 3-level fixation seems more reliable.

The most commonly used surgical technique for patients with ASCF seems to be the posterior approach. The posterior-alone approach is sufficiently stable only if the anterior column of the spine is complete and able to withstand the load, such as in the case of a linear fracture in the axial plane of the vertebral body. Clinical studies have demonstrated a lower failure rate of internal fixation with posterior approaches than with stand-alone anterior plating^58–61^. Fifty percent of ASCF patients have been reported to choose the posterior approach alone^57^. The first advantage of a posterior approach is that a multisegmental laminectomy and decompression can be relatively easy to perform at the same time in the presence of epidural hematoma or neurological deficit. Moreover, with a posterior approach, it is easier to add fixation points for extending the operative segment and further decrease the load arm acting on the fractured site. From the perspective of the RMCs in this study, the 1-level posterior-alone approach is not superior to the anterior approach in terms of flexion and extension resistance, but long segments of posterior fixation have significantly better stability than segments of anterior-alone fixation. Therefore, for ASCF patients who cannot undergo ASF, such as those with excessive cervical kyphosis, long-segment PSF could be a feasible alternative.

However, long posterior fixation involves at least 3 segments and a generally higher level of invasiveness; additionally, the lack of anatomical markers and poor imaging visualization require clear consideration in cases of ASCF. Meanwhile, the problems of anterior decompression and additional external fixation after surgery have not yet been effectively resolved. In addition, poor stabilization of the AS fracture site is a risk factor for secondary spinal cord injury during transition of the surgical position after anesthesia. For these reasons, a posterior-alone approach may not always be recommended.

Considering the fracture mechanism, ASCF often occurs at sites of pathological ossification, and in most cases, these fractures can be classified as three-column fractures. Some researchers have suggested that combined fixation approaches should be used to stabilize the spine^10^, and 25% of patients were treated with combined anterior and posterior approaches, as reported by Westerveld^57^. It is doubtless that 360° spinal fusion and concurrent anterior/posterior decompression can be achieved only through combined approaches. More importantly, a variety of complications may usually be followed by neurological deficits^8,15,62^. Combined surgery can provide strong internal fixation, which allows patients to exercise early for functional recovery and thus reduces the incidence of pulmonary infection associated with long-term bed rest. Although combined approaches are the most invasive option, in cases of ASCF, many studies have shown strong support for this type of treatment^28–32^. This FEM study also found that the combined anterior and posterior approach has the minimal RoM change at the fractured level (C5/6) and the smallest implant MS compared with either approach alone; even 1-level combined fixation provides better stability than 5-level posterior-alone fixation. Considering only biomechanics without the trauma or complications of surgery, combined fixation may be the most stable treatment measure for ASCF.

Based on the fact that the fracture characteristics of ASCF are similar to those of long bone fractures, some researchers have suggested implant lengths approximately three times the diameter of the fractured bone, which is approximately 3 levels long^57^. The results of this study show a general trend that longer segments of fixation provided better stability at the fractured level and lowered the implant MS. However, detailed analysis shows that the stability increase was more significant when the length of posterior-alone fixation was increased from 1 to 3 levels. However, when increasing from 3 to 5 levels, the stability increase rapidly decreased. Thus, 3-level fixation in posterior-alone surgery for ASCF may be the most cost-effective option. In addition, in terms of rod stress, increasing the fixation length does not significantly decrease the stress concentration of the rod in posterior-alone surgery. However, when combined with anterior fixation, the MS was decreased by at least 60% (P1 to A1P1 under extension). Therefore, in the case of severe dislocation and kyphosis, a significant stress concentration on the fracture segment may lead to rod breakage even if the posterior fixation length is increased; another anterior surgery might be performed to avoid rod breakage or fixation failure.

The concept of skipped screwing has been proposed in previous studies and is mainly applied in cases of long bone fracture and spinal deformity. The degree of proximal junctional kyphosis determines whether the implant density is increased in surgery for deformity in adults^63^. The results show that the C5/6 screws in posterior fixation have the largest stress concentration in both consecutive and skipped screwing. This indicates that screws are most likely to fail in the vertebral body adjacent to the fractured segment. Therefore, to provide adequate holding force, these adjacent screws must be placed correctly, especially in skipped screwing, which involves fewer screws. Aside from screw misplacement, the cost of spinal surgery could be increased by the cost of extra screws^64^. The FEM results did not demonstrate a significant difference in the MS and RMC between skipped and consecutive screwing. Considering the cost and risk of each additional screw, long segments of skipped-screw posterior fixation may be an alternative option for patients with ASCF.

The current study has certain limitations. The simplification of the AS spine^65^ is a major limitation, which is detailed, as follows: (1) the stress shield and yield of bone were neglected. Both bone and implants were defined as elastic materials, and fractures were not allowed. (2) Our study is insufficient to investigate stability under fatigue loading, and some cases of implant failure may occur under multicyclic loading but not in single impulse loading for more realistic situations^66^. (3) The FEM in this study has insufficient resolution to simulate and analyze screw pull-out^67^. (4) This model was adjusted based on current knowledge and experience, but no biomechanical verification was performed for the existing AS model^68^. Furthermore, loads with highly idealized conditions cannot comprehensively represent the daily activities of the AS subaxial cervical spine. Last, there are many other factors that need to be considered in clinical research, such as medical complications, surgical tolerance, and the presence and degree of deformity before injury. The role played by biomechanical factors in decision-making remains to be further verified in clinical practice. Several clinical as well as finite element studies^69,70^ have indicated that longer segments of fixation adversely affect adjacent discs and increase disc pressure^71–74^, which increases the risk of adjacent segment disease. Although it does not cause adjacent segment disease (ASD) in AS patients, it may cause stress fractures in adjacent segments. Our study evaluated the stability of ASCF treated with internal fixation from only a biomechanical perspective. The value of the findings for clinical application needs to be verified by large-sample clinical studies. Additionally, the role played by biomechanical factors in decision-making remains to be further verified in clinical practice.

The reader should always keep in mind that it is the assumptions that lead to the presented results. The presented results of the FEM study indicate a trend rather than a precise value due to the simplifications concerning the contact behavior, material properties, tissue geometry and applied loads. However, the current simulated results should be able to support our hypothesis.

## Conclusion

This study adjusted the FEM of C2-T1 from a previously validated model to simulate an AS subaxial cervical fracture. The results provide evidence for clinical decision-making in the surgical treatment of ASCF. Longer fixation seems to be more stable with the anterior/posterior approach alone, but 3-level posterior fixation may be the most cost-effective option. Considering only biomechanics without the trauma or complications of surgery, it is recommended to perform surgery with combined approaches (even 1-level combined fixation). Long skipped-screwing posterior fixation is an alternative method for ASCF due to the similar stability as fixation with consecutive screwing.

## Data Availability

The datasets generated and analyzed during the present study are available from the corresponding author on reasonable request.
